# Supplementary material for: putzig contributes to Piwi-mediated repression of transposon activity in the female germline of Drosophila in a two-tiered fashion to safeguard genome integrity
Source: Cell Mol Life Sci. 2026 Nov 4;83(1):410. doi: 10.1007/s00018-026-06465-x (PMC13740440; doi:10.1007/s00018-026-06465-x)
Supplement: Supplementary file 1 [file 18_2026_6465_MOESM1_ESM.docx]

| **Reagent or Resource** | **Source** | **Identifier** |
| --- | --- | --- |
| **Antibodies and Dyeing reagents** | | |
| Goat anti-Mouse Alkaline Phosphatase | Jackson ImmunoResearch Laboratories | Cat# 115-055-003; RRID: AB_2338528  (1:1000 WB) |
| Goat anti-Rabbit Alkaline Phosphatase | Jackson ImmunoResearch Laboratories | Cat# 111-055-003; RRID: AB_2337947  (1:1000 WB) |
| Goat anti-Rat Alkaline Phosphatase | Jackson ImmunoResearch Laboratories | Cat# 112-055-003; RRID: AB_2338148  (1:1000 WB) |
| Goat anti-Mouse Cy3 | Jackson Immuno Research | Cat# 115-165-166; RRID: AB_2338692 (1:250 IHC) |
| Goat anti-Rabbit Cy3 | Jackson Immuno Research | Cat# 111-165-144; RRID: AB_2338006 (1:250 IHC) |
| Goat anti-Rat Cy3 | Jackson Immuno Research | Cat# 112-165-167; RRID: AB_2338251 (1:250 IHC) |
| Goat anti-Mouse FITC | Jackson Immuno Research | Cat# 115-095-166; RRID: AB_2338601 (1:250 IHC) |
| Goat anti-Guinea Pig Alexa 647 | Jackson Immuno Research | Cat# 106-605-003; RRID: AB_2337446 (1:250 IHC) |
| Guinea Pig anti-Pzg | own group | Kugler and Nagel, 2007 [45]  (1:500 IHC, WB; 1:33.3 for IP) |
| Mouse anti-Ago | Brennecke lab | Brennecke *et al.*, 2007 [8]  (1:50 IHC) |
| Mouse anti-Aub | Brennecke lab | Brennecke *et al.*, 2007 [8]  (1:50 IHC) |
| Mouse anti-Flag M2 | Sigma-Aldrich | Cat# F3165; RRID: AB_259529 (1:1000 WB) |
| Mouse anti- γH2AV | DSHB | Cat# UNC93-5.2.1: RRID: AB_2618077 (1:2000 IHC) |
| Mouse anti-Hts | DSHB | Cat# 1B1: RRID:  AB_528070 (1:20 IHC) |
| Mouse anti-HP1a | DSHB | Cat#C1A9; RRID:AB_528276 (1:50 WB) |
| Mouse anti-Lamin C | DSHB | Cat# lc28.26, RRID:  AB_528339 (1:20 IHC) |
| Mouse anti-Piwi | Brennecke lab, Vienna, Austria | Brennecke *et al.*, 2007 [8]  (1:50 IHC) |
| Mouse anti-Piwi | Santa Cruz Technology | Cat#sc-390946; RRID: AB_1125916 (1:200 WB) |
| Mouse anti-V5 | ChromoTek GmbH | Cat#v5ab; RRID:AB_2868500 (1:1000 WB) |
| Normal Goat Serum (NGS) | Jackson ImmunoResearch Laboratories | Cat# 005-000-121; RRID: AB_2336990 |
| Rabbit anti-Di-Methyl-Histone H3 (Lys4) (C64G9) | Cell Signaling Technology | Cat#9725; RRID:  AB_10205451 (1:500 IHC) |
| Rabbit anti-GFP | ChromoTek GmbH | Cat# pabg1; RRID: AB_2749857 (1:250 WB) |
| Rabbit anti-GFP XChIP grade | Thermo Fisher Scientific | Cat# A-11122; RRID:  AB_221569 (10 μg per XChIP) |
| Rabbit anti-HA | NEB Biolabs | Cat#3724; RRID: AB_1549585 (1:100 IHC) |
| Rabbit anti-H2A | Glaser lab, New York, USA | Leach *et al.*, 2000 (1:2000 WB) |
| Rabbit anti-γH2AX (pSer139) | Trevigen, Inc./Novus | Cat# NB100-384; RRID:  AB_10002815 (1:1000 WB) |
| Rabbit anti-LexA | BioAcademia | Cat#61-001; (1:3000 WB) |
| Rabbit anti-Lsd1 | Di Stefano lab, Toulouse, France | Di Stefano *et al.*, 2007 [32] (1:1000 WB) |
| Rabbit anti-PH3 | Cell Signaling Technology | Cat#9701; RRID:  AB_331535 (1:50 IHC) |
| Rabbit anti-phospho-Smad2 (Ser465/467) | Cell Signaling Technology | Cat#3108; RRID:  AB_490941 (1:50 IHC) |
| Rat anti-HA clone 3F10 | Roche | Cat#11867423001: RRID: AB_390918 (1:500 WB) |
| Rat anti-Pzg | own group | Kugler and Nagel, 2007 [45]  (1:500 WB) |
| Rat anti-Vasa | DSHB | Cat# anti-Vasa; RRID:  AB_760351 (1:50 IHC) |
| Vectashield Mounting Medium | Vector Laboratories | Cat# H-1000; RRID:  AB_2336789 |
| Wheat Germ Agglutinin (WGA), Alexa Fluor^TM^ 647 conjugate | Thermo Fisher Scientific | Cat#W32466 |
| **Experimental Models: *Drosophila melanogaster* strains** | | |
| **Tagged strains/Reporter/mutant and control** | | |
| *w;;****armi****_EGFP/TM3, Ser* | Vienna Drosophila Resource Center | VDRC (JB-stock)  313238 |
| *w; EGFP_****aub*** *[attP40];;* | Vienna Drosophila Resource Center | VDRC (JB-stock)  313244 |
| *w;; 3xFLAG/V5/Precission/GFP_****Cuff*** *[attP2]/TM3, Sb;* | Vienna Drosophila Resource Center | VDRC (JB-stock)  313269 |
| *w;; 3xFLAG/V5/Precission/GFP_****Del*** *[attP2]/TM3, Sb;* | Vienna Drosophila Resource Center | VDRC (JB-stock)  313271 |
| *w;; GFP_****Gasz*** *[attP2]/TM3, Sb;* | Vienna Drosophila Resource Center | VDRC (JB-stock)  313277 |
| *w; 3xFlag/GFP_****mael*** *[attP40]/CyO;;* | Vienna Drosophila Resource Center | VDRC (JB-stock)  313298 |
| *w; ; 3xFLAG/V5/Precission/GFP_CG12721 (****moon****); hs-line [attP2]/TM3,Sb;* | Vienna Drosophila Resource Center | VDRC (JB-stock)  313251 |
| *w;* ***Nup54*** *mCherry/3xHA/Precission/4xMYC [attP40]/CyO;;* | Vienna Drosophila Resource Center | VDRC (JB-stock)  313304 |
| *;; FLAG_V5_GFP_****Nxf2*** *m11-1* | Vienna Drosophila Resource Center | VDRC (JB-stock)  313984 |
| *w;* ***Papi****_GFP_Precission_V5_3xFLAG [attP40]/CyO;Sb/TM3, Ser;* | Vienna Drosophila Resource Center | VDRC (JB-stock)  313715 |
| *w;; EGFP_Piwi [attP2]* | Vienna Drosophila Resource Center | VDRC (JB-stock)  313319 |
| *;; FlyFos023014(pRedFlp-Hgr)(****Z4^18261^****::2XTY1-SGFP-V5-preTEV-BLRP-3XFLAG)dFRT =* ***pzg_GFP*** | Vienna Drosophila Resource Center | VDRC 318536 |
| *w; AID_3xFLAG_V5_Precission_GFP_****rhino****/CyO;;* | Vienna Drosophila Resource Center | VDRC (JB-stock)  313993 |
| *;;EGFP-****rhi*** *[attP2]* | Vienna Drosophila Resource Center | VDRC (JB-stock)  313340 |
| *pNASA > LAP*  ***TRF2s****[attP40]/CyO* | Vienna Drosophila Resource Center | VDRC (JB-stock)  313774 |
| *w; EGFP_****vas*** *[attP40];;* | Vienna Drosophila Resource Center | VDRC (JB-stock)  313368 |
| *w;;* ***zuc****_GFP_Precission_V5_3XFLAG [attP2] /TM3,Sb;* | Vienna Drosophila Resource Center | VDRC (JB-stock)  313656 |
| *w;P{NRE-EGFP.S}1* | Bloomington stock center | BL30728;  RRID:BDSC_30728 |
| *P{RFP-HP1}3* | Bloomington stock center | BL30562;  RRID:BDSC_30562 |
| *P53R-GFPnls* | J. Abrams | Lu *et al.*, 2010 |
| *nos>NLS_GFP_lacZ_vas-3’UTR_burdock-target [attP2] /TM3,Sb* | Vienna Drosophila Resource Center | VDRC (JB-stock)  313212 |
| *Het-A-lacZ* |  | Shpiz *et al*., 2007 |
| *gypsy-lacZ/CyO* | Vienna Drosophila Resource Center | VDRC (JB-stock)  313222 |
| *pzg^66^/TM6B, Ubi-GFP, Tb* | own group | Kugler *et al.*, 2011 [46] |
| *y^1^w^67c23^* | Bloomington stock center | BL6599:  RRID:BDSC_6599 |
| **Gal4/UAS lines, hs-line** |  |  |
| *matα*-Gal4: *y^1^ w*;* P{*matalpha4-*GAL-VP16}67; P{*matalpha4*-GAL-VP16}15 | Bloomington stock center | BL80361;  RRID:BDSC_80361 |
| *nos-*Gal4: *w;; P{w^+mC^=GAL4::VP16-nanos.UTR}CG6325^MVD1^* | Bloomington stock center | BL4937;  RRID:BDSC_4937 |
| *w;P{w^+mC^=GAL4::VP16-nanos.UTR}CG6325^MVD1^, P{w^+mC^=UASp-GFPS65C-alphaTub84B}3* | Bloomington stock center | BL7253;  RRID:BDSC_7253 |
| *bab-*Gal4: *w^*^;; P{w^+mW.hs^=GawB}bab1^Agal4-5^/TM3, Sb^1^* | Bloomington stock center | BL6802;  RRID:BDSC_6802 |
| *c587-*Gal4: *P{w^+mW.hs^=GawB}C587, w^*^;;* | Bloomington stock center | BL67747;  RRID:BDSC_67747 |
| UAS-shRNA-*Dref: y^1^ sc^*^ v^1^ sev^21^; P{y^+t7.7^ v^+t1.8^=TRiP.GL00532}attP2/ TM3, Sb^1^* | Bloomington stock center | BL36865;  RRID:BDSC_36865 |
| UAS-shRNA-*foxo: y^1^ sc^*^ v^1^ sev^21^;; P{y^+t7.7^ v^+t1.8^=TRiP.HMS00422}attP2;* | Bloomington stock center | BL32427;  RRID:BDSC_32427 |
| UAS-shRNA-*Nurf301: y^1^ sc^*^ v^1^ sev^21^; P{y^+t7.7^ v^+t1.8^=TRiP.GL00265}attP2* | Bloomington stock center | BL35353;  RRID:BDSC_35353 |
| UAS-shRNA-*pzg*: *y^1^ sc^*^ v^1^ sev^21^;; P{y^+t7.7^ v^+t1.8^=TRiP.GL00373}attP2;* | Bloomington stock center | BL35448;  RRID:BDSC_35448 |
| *UAS-shRNA-Tom: y^1^ sc^*^ v^1^ sev^21^;;*  *P{y^+t7.7^ v^+t1.8^=TRiP.HMS05421}attP40* | Bloomington stock center | BL66955;  RRID:BDSC_66955 |
| *UAS-shRNA-w: y^1^ v^1^; ;*  *P{y^+t7.7^ v^+t1.8^=TRiP.HMS00004}attP2/TM3, Sb^1^* | Bloomington stock center | BL33613;  RRID:BDSC_33613 |
| UASp-*lacZ*: *w^+mC,^ P{UASp-lacZ}* | P. Rørth | Rørth, 1998 |
| UASt-*Akt1: y^1^ w^1118^; P{w^+mC^=UAS-Akt.Exel}2* | Bloomington stock center | BL8191;  RRID:BDSC_8191 |
| UASt-*mir125* | L. Johnston | Caygill and Johnston, 2008 |
| UASt-*Rheb: y^1^ w^*^ hs-flp;; P{EP(loxP.UAS)y^+^.UAS}RhebEP^50.084^* | E. Hafen | Stocker *et al.*, 2003 |
| UASt-*S6K^TE^*: w; *P{w^+mC^=* UAS-*S6K^TE^}2* | Bloomington stock center | BL6912;  RRID:BDSC_6912 |
| *hs-N^intra^* | S. Artavanis-Tsakonas | Lieber *et al.*, 1993 [78] |
| **Yeast constructs and vectors** | | |
| pEG202 | E. Golemis | Gyuris *et al.*, 1993 [57] |
| pEG-*armi* (3.6 kb *armi* *EcoRI*/*NcoI*) | This work | N/A |
| pEG-*cuff* (1.2 kb *cuff BamHI*/*XhoI*) | This work | N/A |
| pEG-*del* (3 kb *del EcoRI*/*XhoI*) | This work | N/A |
| pEG-*gasz* (1.4 kb *gasz EcoRI*/*XhoI*) | This work | N/A |
| pEG-*Hp1a* (0.6 kb *Hp1a* *EcoRI*/*XhoI*) | This work | N/A |
| pEG-*lsd1* (2.7 kb *lsd1 BglII*/*XhoI*) | This work | N/A |
| pEG-*mael* (1.4 kb *mael BamHI*/*XhoI*) | This work | N/A |
| pEG-*moon* (0.5 kb *moon EcoRI*/*XhoI*) | This work | N/A |
| pEG-*nxf2* (2.6 kb *nxf2 EcoRI*/*NcoI*) | This work | N/A |
| pEG-*nup54* (1.9 kb *nup54 EcoRI*/*XhoI*) | This work | N/A |
| pEG-*papi* (1.8 kb *papi EcoRI*/*NotI*) | This work | N/A |
| pEG-*piwi* (2.6 kb *piwi BglII*/*NotI*) | This work | N/A |
| pEG-*rhi* (1.3 kb *rhi EcoRI*/*XhoI*) | This work | N/A |
| pEG-*trf2* (1.9 kb *trf2 BamHI/ XhoI*) | This work | N/A |
| pEG-*zuc* (0.8 kb *zuc NcoI*/*XhoI*) | This work | N/A |
| pJG4-5 vector | E. Golemis | Vojtek *et al.*, 1993 |
| pJG-Pzg-FL (3kb *Pzg* *EcoRI*/*XhoI*) | This work | N/A |
| pJG-*pzg*-N (1.6 kb *pzg EcoRI*/*XhoI; AS 1-543*) | This work | N/A |
| pJG-*pzg*-C (1.4 kb *pzg EcoRI*/*XhoI; AS 544-996*) | This work | N/A |
| psH18-34 | E. Golemis | Gyuris *et al.*, 1993 [57] |
| **Critical Commercial kit systems and chemicals** | | |
| Click-iT^®^ EdU Alexa Fluor 555 Imaging Kit for EdU staining | Invitrogen | Cat#C10338 |
| DNA Screen Tape Analysis D1000 Screen Tape and Reagents | Agilent Technologies | Cat # 5067-5582/83 |
| Dynabeads^TM^ Protein G | Thermo Fisher Scientific | Cat# 1003D |
| Protein A magnetic beads | New England Biolabs | Cat#S1425S |
| Binding Control Magnetic Agarose | ChromoTek GmbH | Cat#bmab; RRID: AB_2827548 |
| GFP-Trap^®^ Magnetic Agarose | ChromoTek GmbH | Cat#gtma; RRID: AB_2631358 |
| Myc-Trap^®^ Magnetic Agarose | ChromoTek GmbH | Cat#ytma; RRID: AB_2631370 |
| RFP-Trap^®^ Magnetic Agarose | ChromoTek GmbH | Cat#rtma; RRID: AB_2631363 |
| NextSeq 500/550 Mid-Output v2.5 Kit | Illumina | Cat#20024904/5 |
| Qubit 1X dsDNA HS Assay Kit | Thermo Fisher Scientific | Cat#Q33230 |
| Lamivudine, 3TC | Sigma-Aldrich | Cat#PHR1365 |
| Miracloth filter | Millipore | Cat# 475855 |
| Protease inhibitor cocktail | Roche | Cat# 05892791001 |
| Magnetic Separation Rack | New England Biolabs | Cat# S1506S |
| Universal-nuclease | Pierce | Cat# 88701 |
| **Oligonucleotides for cloning of pEG/pJG yeast constructs, 5‘-3‘orientation** | | |
| Armi_UP_*EcoRI:* ATTTGAATTCATGTTCACATACGTTAGCAAG | Microsynth | N/A |
| Armi_LP_*NcoI:* TCATCCATGGTTAGTTCAAATC ATCTGTAGTA | Microsynth | N/A |
| Boot_UP_*BamHI:* CGAGGGATCCCGATGACG GATTTCAATGCATC | Microsynth | N/A |
| Boot_LP_*NcoI:* TGTCCCATGGCTAGTTAAA  ACGTTGCAGTTGG | Microsynth | N/A |
| Cuff_UP_Bam*HI:* CGTTGGATCCAGATGAAT TCTAATTACACAATAT | Microsynth | N/A |
| Cuff_LP_Xho*I:* ATTGCTCGAGTTAAACTATAGAAGACATGGTT | Microsynth | N/A |
| Del_UP_*EcoRI:* TGGCGAATTCATGGAAAAGTTGGACA | Microsynth | N/A |
| Del_LP_Xho*I:* TTTCCTCGAGTTAATCAAAATTATGTATATTG | Microsynth | N/A |
| Gasz_UP_*EcoRI:* AAGTGAATTCATGATGAGCAACCTTTG | Microsynth | N/A |
| Gasz_LP_Xho*I:* TGAGCTCGAGCTATGAGAACCACTTGC | Microsynth | N/A |
| HP1a_UP_*EcoRI*:  CATAGAATTCATGGGCAAGAAAATCGACAACC | Microsynth | N/A |
| HP1a_LP_*XhoI*:  GTACCTCGAGTTAATCTTCATTATCAGAGTACCAGGA | Microsynth | N/A |
| Lsd1_UP_*BglII:* CATAAGATCTACATGAAACCCACCCAGTT | Microsynth | N/A |
| Lsd1_LP_Xho*I:* AAATCTCGAGTTACTGTAGCTCCGTAG | Microsynth | N/A |
| Mael_UP_*BamHI:* ACGCGGATCCAGATGGCTCCTAAGAAGCATAG | Microsynth | N/A |
| Mael_LP_ Xho*I :* CCAACTCGAGTTATTTTTTAAGTTTCCCATCA | Microsynth | N/A |
| Moon_UP_ *EcoRI:* TGGCGAATTCATGGCCAAGATATTGCC | Microsynth | N/A |
| Moon_LP_ Xho*I:* GCTGCTCGAGCTACATTTCCTGGCCTG | Microsynth | N/A |
| Nup54_UP_ *EcoRI:* ATACGAATTCATGTCGTTCTTCGGATCCAACA | Microsynth | N/A |
| Nup54_LP_ Xho*I:* TCCACTCGAGTCACGATTGTCGCAGCTCGGGC | Microsynth | N/A |
| Papi_UP_EcoR*I:* GTTAGAATTCATGTTGCGCAACACGCCTTTCG | Microsynth | N/A |
| Papi_LP_Not*I :* CAATGCGGCCGCCTAATGCGCGCTAGCACCATTT | Microsynth | N/A |
| Piwi_UP_Bgl*II:* CAACAGATCTTAATGGCTGATGATCAGGGACG | Microsynth | N/A |
| Piwi_LP_Not*I:* AATTGCGGCCGCTTATAGATAATAAAACTTCTTTTCG | Microsynth | N/A |
| Pzg_UP_*EcoR*I: ACACGAATTCATGAACAACCAACTGAACCCG | Microsynth | N/A |
| Pzg_LP_AS220_*XhoI*: TAATCTCGAGTTAGGTTCCTGGGGCGATCTTGGAC | Microsynth | N/A |
| Pzg_UP_AS221_*EcoRI*: ATTAGAATTCACCCAGAGCAGCGTCCAAGGCA | Microsynth | N/A |
| Pzg_1967_LP_*XhoI*: TATTCTCGAGTTACTCTCCGGGCTCATCGCTTA | Microsynth | N/A |
| Pzg_1970_UP_*EcoRI*: TTTAGAATTCGCAACTGGTAAGGCCGGTGAC | Microsynth | N/A |
| Pzg_LP_*XhoI*: CGGTCTCGAGCTAGTCGGTCTTTGTCTCTGTAAACT | Microsynth | N/A |
| Rhi_UP_EcoR*I:* CGGAGAATTCATGTCTCGCAACCATCA | Microsynth | N/A |
| Rhi_LP_Xho*I:* CTAGCTCGAGTTACTTGGGCACAATGATCCTCA | Microsynth | N/A |
| Trf2_RH_UP_*BamHI*:  TCATGGATCCTTATGCAAAACGATATGGTCAGCATAC | Microsynth | N/A |
| Trf2_RH_LP_*XhoI*:  GCTACTCGAGTTAGAAGGGCATATCCAATTCGTTG | Microsynth | N/A |
| Zuc_UP_*NcoI*: AATTCCATGGATGTTGATTACCCAAATAATT | Microsynth | N/A |
| Zuc_LP_Xho*I*: CAATCTCGAGCTACTTGAGCTGGATTT | Microsynth | N/A |
| **Oligonucleotides: genotyping** |  |  |
| Akt1_internally: CCTTTCGATTACCGTGGTCCATTG | Microsynth | N/A |
| eGFP_UP: GGTACGCGCTAGAGTCGAGAG | Microsynth | N/A |
| eGFP_LP: GTGGTATGGCTGATTATGATCTAGAG | Microsynth | N/A |
| Gal4_LP: AAGATGTAGGGCTGTCACCAA | Microsynth | N/A |
| Gal4VP16_UP: GCTGACTAGGGCACATCTGACA | Microsynth | N/A |
| Gal4VP16_LP: CATATCCAGAGCGCCGTAGG | Microsynth | N/A |
| GFP_UP: GCCACAAGTTCAGCGTGTCCG | Microsynth | N/A |
| GFP_LP: TCACCTTGATGCCGTTCTTCTGC | Microsynth | N/A |
| lacZ_UP: CAACGTCGTGAGTGGGAAAACC | Microsynth | N/A |
| lacZ_LP: CCCGTTGCACCACAGATGAAA | Microsynth | N/A |
| Notchintra_UP: CGCCCGTTTTATCCATTGTG | Microsynth | N/A |
| Hsp70_LP: ACGGAGGGACAATTCAATTCAA | Microsynth | N/A |
| pUASt_5‘: CGCACAGTCACGTTATTG | Microsynth | N/A |
| P-end_5‘: CGACGGGACCACCTTATG | Microsynth | N/A |
| pValium20_UP: ACCAGCAACCATCAAC | Microsynth | N/A |
| pValium20_LP: TAATCGTGTGTGATGCCTACC | Microsynth | N/A |
| pValium22_UP: GGTGATAGAGCCTGAACCAG | Microsynth | N/A |
| pValium22_LP: TAATCGTGTGTGATGCCTACC | Microsynth | N/A |
| Rheb_internally_LP‘: TTCTCCTGGGGATTGCCGTTCT | Microsynth | N/A |
| **Stellaris™ FISH probes, all marked with Quasar® 570** | | |
| **38C2 set (5‘-3‘ orientation)** |  |  |
| Cluster5_1 TATTTTAATGTGCGCGGTACG | LGC Technologies, Inc. (Hoddesdon, UK) | N/A |
| Cluster5_2 GTCGCTTAATTCAACTTTTCC | LGC Technologies, Inc. (Hoddesdon, UK) | N/A |
| Cluster5_3 CTTCAGTTAGTTCTATTCCTA | LGC Technologies, Inc. (Hoddesdon, UK) | N/A |
| Cluster5_4 TCTTATCTCTTTCTCATTCAT | LGC Technologies, Inc. (Hoddesdon, UK) | N/A |
| Cluster5_5 ATCGCATCCTTTGTATTATTT | LGC Technologies, Inc. (Hoddesdon, UK) | N/A |
| Cluster5_6 CGCACGCGCTTAAATATTTAA | LGC Technologies, Inc. (Hoddesdon, UK) | N/A |
| Cluster5_7  GCTTGGTCGAGTGTGACC | LGC Technologies, Inc. (Hoddesdon, UK) | N/A |
| Cluster5_8 CCACACTAATACAGGCAAATG | LGC Technologies, Inc. (Hoddesdon, UK) | N/A |
| Cluster5_9  GACTGGGATTAGTGTGCC | LGC Technologies, Inc. (Hoddesdon, UK) | N/A |
| Cluster5_10  CGCGCGCTTTATATAGGC | LGC Technologies, Inc. (Hoddesdon, UK) | N/A |
| Cluster5_11 AAATACCGACTGGCTAATGCA | LGC Technologies, Inc. (Hoddesdon, UK) | N/A |
| Cluster5_12 ATATGACTTCGCTGCTGACTA | LGC Technologies, Inc. (Hoddesdon, UK) | N/A |
| Cluster5_13 CAATAGACTCGGATTTCAGGA | LGC Technologies, Inc. (Hoddesdon, UK) | N/A |
| Cluster5_14 CTCCATCAGAGCATTTTATAC | LGC Technologies, Inc. (Hoddesdon, UK) | N/A |
| Cluster5_15 ATCATTTTCCAAATTCGTGCA | LGC Technologies, Inc. (Hoddesdon, UK) | N/A |
| Cluster5_16 GGCCAATAAAGTTCGATTCGA | LGC Technologies, Inc. (Hoddesdon, UK) | N/A |
| Cluster5_17 GCGAGTCAGATGATGATGTTA | LGC Technologies, Inc. (Hoddesdon, UK) | N/A |
| Cluster5_18  GGTACTTGCAGAAATCCT | LGC Technologies, Inc. (Hoddesdon, UK) | N/A |
| Cluster5_19 CCTCCTGAAAATATATTTGCG | LGC Technologies, Inc. (Hoddesdon, UK) | N/A |
| Cluster5_20 AACTAGTAGAGTTAATCCCTG | LGC Technologies, Inc. (Hoddesdon, UK) | N/A |
| Cluster5_21 AGGATCACACAGACTTTTTCT | LGC Technologies, Inc. (Hoddesdon, UK) | N/A |
| Cluster5_22 GCAAAAGTGGAACAGCATCAC | LGC Technologies, Inc. (Hoddesdon, UK) | N/A |
| Cluster5_23 TCCACTTCGACATATTCTTAA | LGC Technologies, Inc. (Hoddesdon, UK) | N/A |
| Cluster5_24 GGGCACAAATACAGGATGGAA | LGC Technologies, Inc. (Hoddesdon, UK) | N/A |
| Cluster5_25 AGTTAGGTTGGAATCCAAGAC | LGC Technologies, Inc. (Hoddesdon, UK) | N/A |
| Cluster5_26 CAGTGCCATAAAAACCCGAAA | LGC Technologies, Inc. (Hoddesdon, UK) | N/A |
| Cluster5_27 AAATGCTGTAAGATCACCTCC | LGC Technologies, Inc. (Hoddesdon, UK) | N/A |
| Cluster5_28 AGTTGCAATAATGCCGATATT | LGC Technologies, Inc. (Hoddesdon, UK) | N/A |
| Cluster5_29 CCAGTTGATATTGGGGTTAAA | LGC Technologies, Inc. (Hoddesdon, UK) | N/A |
| Cluster5_30 TTTATCACTGTTTTTATGCCA | LGC Technologies, Inc. (Hoddesdon, UK) | N/A |
| Cluster5_31 GATGGGGGGAAAAATGCCAAT | LGC Technologies, Inc. (Hoddesdon, UK) | N/A |
| Cluster *42AB* probes | LGC Technologies, Inc. (Hoddesdon, UK) | Andersen *et al.*, 2017 [12] |
| **Software, Algorithms and Imaging systems** | | |
| Axioskop 2 Plus coupled with Canon EOS 700D camera | Carl Zeiss/Canon | N/A |
| Axiophot stereomicroscope coupled with Pixera PVC 100C camera | Carl Zeiss/Pixera Corporation | N/A |
| MRC1024 Laser scanning confocal imaging system and Axiophot microscope | Bio-Rad/Zeiss | N/A |
| LSM700 confocal microscope | Carl Zeiss Microscopy | N/A |
| 3i W1 Spinning Disk microscope | 3i Intelligent Imaging Innovations GmbH | N/A |
| SlideBook 2025.2.0.47644 software for 3i W1 Spinning Disk microscope | 3i Intelligent Imaging Innovations GmbH | N/A |
| LaserSharp 2000 software for MRC1024 | Bio-Rad | N/A |
| Creation of images using LSM700 confocal microscope | ZEN  Version SP7 | Carl Zeiss Microscopy GmbH |
| Figure assembly | CorelDRAW® Graphics Suits Version 9 | RRID:SCR_014235 |
| Picture processing and analyzing | Corel Photo Paint® Version 9.337 | N/A |
| Data processing and analyzing | Fiji (Image J2)  Version 2.16.0/1.54p | Schindelin *et al.*, 2012; RRID:SCR_003070 [53] |
| Image deconvolution | DeconvolutionLab2 plugin  Richardson-Lucy algorithm | Sage *et al.*, 2017 [54]  Richardson, 1972; Lucy, 1974 |
| Filtering ChIP-Seq data | BBMap  Version 39.19 | Bushnell B. (sourceforge.net/projects/bbmap/) |
| Intersection with blacklist regions | Bedtools  Version 2.31.0 | Quinlan, 2014 |
| ChIP-seq Alignment | Bowtie2  Version 2.5.4 | Langmead and Salzberg, 2012 |
| Use of various tools for ChIP-seq data processing and analysis | deepTools  Version 3.5.6 | Ramírez *et al.*, 2016 |
| ChIP-seq Quality analysis | FastQC  Version 0.12.1 | Andrew S. (www.bioinformatics.babraham.ac.uk/projects/fastqc/) |
| Visualizing of ChIP-seq alignments | IGV  Version 2.18.4 | Robinson *et al*., 2011 |
| Removal of ChIP-seq duplicates | Picard  Version 3.3.0 | Broad Institute, 2019. Picard Toolkit. Broad Inst. GitHub Repos |
| Use of various tools for ChIP-seq data processing | SAMtools  Version 1.21 | Danecek *et al.*, 2021 |
| Creation of ChIP-seq fragmentation profiles | TapeStation Analysis Software  Version 4.1.1 | Agilent Technologies |
| ChIP-seq quality analysis and data processing | Trim Galore  Version 0.6.10 | Krueger F. (www.bioinformatics.babraham.ac.uk/  Projects/trim:galore/) |
| Data analysis and visualization | R  Version 4.5.1 | R Core Team, 2024 (https://www.R-project.org/) |
| User interface for R | R-studio  Version 2025.05.0+496 | Posit team, 2025 (http://www.posit.co/) |
| Creation of diagrams in R | dplyr  Version 1.1.4  ggplot2  Version 3.5.2  tidyverse  Version 2.0.0 | Wickham *et al.*, 2025: dplyr: https://dplyr.tidyverse.org  Wickham, 2016  Wickham *et al.*, 2019 |
| IP-MS data analysis | MaxQuant  Version 2.6.7.0 | Cox and Mann, 2008 |
| Statistical evaluation of IP-MS data | Perseus  2.0.11 | Tyanova *et al.*, 2016 |
| GO analyses of IP-MS data | ShinyGO  Version 0.81 | Ge *et al.*, 2020 |
| Workbench version | bms/Biozym  version2.0.7 | Cat # 68MIC-HRM |
| GraphPad Prism | GraphPad Software, LLC  version 11.0.2 | RRID:SCR_002798 |
| Peptide detectability calculation | DeepMSPeptide | Serrano et al., 2020 [93] |
| **Deposited data** | | |
| Mass Spectrometry datasets shown in the paper | This study | PRIDE: PXD075330 |
| Pzg-GFP ChIP-seq data shown in this paper | This study | GEO series GSE324254 |
| Rhi ChIP-seq data | Baumgartner *et al.*, 2024 [82] | GEO series GSE202465 |
| Del ChIP-seq data | Mohn *et al.*, 2014 [11] | GEO series GSE55824 |
| Cuff ChIP-seq data | Mohn *et al.*, 2014 [11] | GEO series GSE55824 |
| **Materials and equipment for qRT-PCR** | | |
| Blue S'Green qPCR Kit (for qPCR in *Drosophila* tissue) | Biozym | Cat # 331416 |
| DNaseI | New England Biolabs | Cat # M0303 |
| Dynabeads^TM^ mRNA DIRECT^TM^ Micro Purification Kit | Invitrogen, Thermo Fisher Scientific | Cat # 61021 |
| miRNeasy Micro Kit (for isolation of total RNA from ovaries) | Qiagen | Cat # 217084 |
| RNase-free DNase set | Qiagen | Cat # 79254 |
| ProtoScript II First strand cDNA synthesis Kit | New England Biolabs | Cat # E6560S |
| qScriber^TM^ cDNA Synthesis Kit | highQu | Cat # RTK0104 |
| RNA*later* Stabilization Solution | Thermo Fisher Scientific | Cat # AM7020 |
| Mic Magnetic Induction Cycler | bio molecular systems/Biozym | Cat # 68MIC-2 |
| **Oligonucleotides used for transposon, controls and *tom* qRT-PCR** | | |
| Blood_UP:  AACAATAGAAAGAAGCCACCGAAC | Microsynth | Handler *et al.*, 2011 |
| Blood_LP:  AGTCATGGACTATTGAGGGTGTTG | Microsynth | Handler *et al.*, 2011 |
| Burdock_UP:  TTGTGCAACCAATCGGCTCTC | Microsynth | N/A |
| Burdock_LP:  ATGAAGTGGAGGTGCGTTCGC | Microsynth | N/A |
| Copia_UP:  CGACAGTGTGGAGGTTGTGCC | Microsynth | Shpiz *et al*., 2011 |
| Copia_LP:  CTTGGAGACGCTTTACGGACAT | Microsynth | Shpiz *et al.*, 2011 |
| CPSF_UP: GGGTTTTCTGGTGACGGGACTTG | Microsynth | N/A |
| CPSF_LP: CGACAATGTGCGATACTCCTCCTG | Microsynth | N/A |
| cyp33_UP: CTCTGCGGACGCACAATTC | Microsynth | PP14577 DRSC FlyPrimerBank |
| cyp33_LP: TGCAACCAGTCGTCATCTGC | Microsynth | PP14577 DRSC FlyPrimerBank |
| dlp_UP: ATGCGGGTAGTGGAGCTGGTTCT | Microsynth | N/A |
| dlp_LP:  GTCCGGCTTATGGCTGGGTTC | Microsynth | N/A |
| Dref_UP: CCTCCAGGCGCACGTTATAT | Microsynth | PD70194 DRSC FlyPrimerBank |
| Dref_LP: CGAACGGTTCGCTCTAGGTT | Microsynth | PD70194 DRSC FlyPrimerBank |
| F-element_UP:  TTGTTGAACAGCATACCACTCC | Microsynth | Czech *et al.*, 2008 |
| F-element_LP:  CCAGAGTTGATGAGCCAGTGTA | Microsynth | Czech *et al.*, 2008 |
| Gal4VP16_1_UP: GAAGTGCGCCAAGTGTCTGAAGAAC | Microsynth | N/A |
| Gal4VP16_1_LP: CTGTCAGATGTGCCCTAGTCAGCG | Microsynth | N/A |
| gypsy_UP:  CCATACCCAGCTATACAGGTGAGATGG | Microsynth | N/A |
| gypsy_LP:  GACCGTTTTGATATTGGGGAACAG | Microsynth | N/A |
| HetA_UP:  CCAGGCAAGCGGACAAACGA | Microsynth | Shpiz *et al.*, 2011 |
| HetA_LP:  GGAGTGATGAGCGGCGGAAA | Microsynth | Shpiz *et al.*, 2011 |
| Hobo_UP:  AAT CGG CGC TAT CTA TGG GGA A | Microsynth | N/A |
| Hobo_LP:  CACTTGCTCTTCCGCTATCCACAG | Microsynth | N/A |
| Hopper_UP: CAAGATCCCGCAGGCAGCAC | Microsynth | N/A |
| Hopper_LP: TGAGTGGCCCAAGACAGCAAGC | Microsynth | N/A |
| Idefix_UP:  ATTCCACAAGACCGTAAACGA AAAG | Microsynth | N/A |
| Idefix_LP:  CATATAGGAAAATGTCTGCTGGAAATC | Microsynth | N/A |
| I-element_UP:  TGAAATACGGCATACTGCCCCCA | Microsynth | Teo *et al.*, 2018 |
| I-element_LP:  GCTGATAGGGAGTCGGAGCAGATA | Microsynth | Teo *et al.*, 2018 |
| jockey_UP:  TATTGGCACTGCTCAGGCTAAA | Microsynth | N/A |
| jockey_LP:  TTTCTTCGAGCAAGGGCCTTAT | Microsynth | N/A |
| LamC_UP: CTGGAGAACCTCTTGGACACGGA | Microsynth | N/A |
| LamC_LP: CCTCACCGCACAGCAGTTTGTC | Microsynth | N/A |
| mdg1_UP:  ATATGTGCGACAAATTTCGTCCAC | Microsynth | Batki *et al.*, 2019 [24] |
| mdg1_LP:  CAAGTATCTTTGTCTTGATTACCTCAAC | Microsynth | Batki *et al.*, 2019 [24] |
| pzg_UP:  GAACCACAACCATCTGACGAAGGAG | Microsynth | N/A |
| pzg_LP: AGTAACCTCGCCTTCGCCAGATT | Microsynth | N/A |
| R2_UP: ATGCTCCCGAAACAACAAAC | Microsynth | Teo *et al.*, 2018 |
| R2_LP: GCACTGCAGACTTGGTTCAA | Microsynth | Teo *et al.*, 2018 |
| roo_UP:  CGTCTGCAATGTACTGGCTCT | Microsynth | Iwasaki *et al.*, 2021 |
| roo_LP:  CGGCACTCCACTAACTTCTCC | Microsynth | Iwasaki *et al.*, 2021 |
| SdhA_UP: GGACGGTGTGAACAAGATGAAGGAG | Microsynth | N/A |
| SdhA_LP: CGCTCACAATAGTCATCTGGGCAT | Microsynth | N/A |
| S-element_UP:  CTGTGCGCCAAGTCATCCTACG | Microsynth | N/A |
| S-element_LP:  CAACGGTTTGCGCCATACTCTG | Microsynth | N/A |
| TART-A1_UP:  AATGAACTTTGTCTGCCCTCCCA | Microsynth | Shpiz *et al.*, 2011 |
| TART-A1_LP:  ATCTGTCTACTGTCCGCCTTCGCTA | Microsynth | Shpiz *et al.*, 2011 |
| tbp_UP: TAAGCCCCAACTTCTCGATTCC | Microsynth | PP1556 DRSC FlyPrimerBank |
| tbp_LP: GCCAAGAGACCTGATCCC | Microsynth | PP1556 DRSC FlyPrimerBank |
| Tom_UP: ACTGGTCAAACCCCTTCTGC | Microsynth | PD44529 DRSC FlyPrimerBank |
| Tom_LP: ATCTTTGCGCATGTCCTCCA | Microsynth | PD44529 DRSC FlyPrimerBank |
| Transib2_UP:  AAACTGCCCGAGTGTAACAGACAAC | Microsynth | N/A |
| Transib2_LP:  TTTCGGCGTCCCATTTGCTG | Microsynth | N/A |
| ZAM_UP:  CTACGAAATGGCAAGATTAATTCCACTTCC | Microsynth | N/A |
| ZAM_LP:  CCCGTTTCCTTTATGTCGCAGTAGCT | Microsynth | N/A |
| 297_UP:  AACCCGGAACATTGCCGAGA | Microsynth | N/A |
| 297_LP:  AAGCTCCGTCTCTGTCTGCC | Microsynth | N/A |
| 412_UP:  AAAGTACGGTCCAATGAAGACG | Microsynth | Czech *et al.*, 2008 |
| 412_LP:  GTGGTGATGAGCTGTTGATGTT | Microsynth | Czech *et al.*, 2008 |
| **Oligonucleotides used for piRNA/controls reverse transcription** | | |
| clFlam_RT_plus:  GGGCAATAGTACGCTAGTCCG | Microsynth | N/A |
| cl20A1_RT_plus:  ATGGGCGATACATTGTGTCAA | Microsynth | N/A |
| cl38C1_RT_plus:  CCT CCT CTT CGG ACT CCT ACG | Microsynth | N/A |
| cl38C1_RT_minus:  CGT CTT TGC CAA GCA CTC C | Microsynth | N/A |
| cl38C2_RT_plus:  CATTTTTCGGCGTCCACAGT | Microsynth | N/A |
| cl38C2_RT_minus:  GTGGGCTGTGATAGCTGATAGTGT | Microsynth | N/A |
| cl42AB_RT_plus:  CGAAGCCTTAGATCTCGCTCC | Microsynth | Klattenhoff *et al.*, 2009 [10] |
| cl42AB_RT_minus:  ACA TCA GGA ACA CAG CGA GGT G | Microsynth | Klattenhoff *et al.*, 2009 [10] |
| cl80F_RT_plus:  TCTCCCTGACCAACACGAAAC | Microsynth | N/A |
| cl80F_RT_minus:  GCAATCTCGCTGGGAGTGAT | Microsynth | N/A |
| CPSF_RT_1:  ATG GCA CTG CAC CCG AAA C | Microsynth | N/A |
| cyp33_RT_1:  CAC AAT TGC CGA AAG TTC TCC | Microsynth | N/A |
| sdha_RT_1:  TGC TTG CGC CAG TGC TGA | Microsynth | N/A |
| tbp_1_RT_1:  CCC ACA CTC CGC TCA CTC A | Microsynth | N/A |
| dlp_RT_1:  GCC TGC ATC CGT GTC CTC | Microsynth | N/A |
| LamC_RT_1:  GAT TCC AGA ATG CCA GGT GTA GC | Microsynth | N/A |
| **Oligonucleotides used for piRNA qRT-PCR** | | |
| clFlam_UP: CTGAGGAATGAATCGCTTTGAAGTA | Microsynth | N/A |
| clFlam_LP: GTAGTATTGCCTACTTTTTAGCCGA | Microsynth | N/A |
| cl20A_UP: GTGTAGCCGGCAAAATGCAGC | Microsynth | N/A |
| cl20A_LP: CCCTCAATATGTAGAGTAGTGCGAG | Microsynth | N/A |
| cl38C1_UP:  GGT ATG AAG ATT GGA GGC GGC TTG | Microsynth | N/A |
| cl38C1_LP:  CCA CCC ACC ACC AAG CTG CTC TA | Microsynth | N/A |
| cl38C2_UP: CTTGGTAACAAGTACCGTATGGTCG | Microsynth | N/A |
| cl38C2_LP: GGACCTTCCCTGCTCCCTACAA | Microsynth | N/A |
| cl42AB_UP:  GCGT CCC AGC CTA CCT AGT CAG AA | Microsynth | N/A |
| cl42AB_LP:  ACT TCC CGG TGA AGA CTC CTT CC | Microsynth | N/A |
| cl80F_UP: TCGTGAGTCGCGTCAACATCCT | Microsynth | N/A |
| cl80F_LP: GAGGCAGTACCTTTCCCGCTCA | Microsynth | N/A |

**References**

Caygill EE, Johnston LA (2008) Temporal regulation of metamorphic processes in *Drosophila* by the let-7 and miR-125 heterochronic microRNAs. Curr Biol 18(13):943-50 https://doi.org/10.1016/j.cub.2008.06.020

Cox J, Mann M (2008) MaxQuant enables high peptide identification rates, individualized p.p.b.-range mass accuracies and proteome-wide protein quantification. Nat. Biotechnol*.* 26 (12):1367-1372. https://doi.org/10.1038/nbt.1511

Czech B, Malone CD, Zhou R, Stark A, Schlingeheyde C, Dus M, Perrimon N, Kellis M, Wohlschlegel JA, Sachidanandam R, Hannon GJ, Brennecke J (2008) An endogenous small interfering RNA pathway in *Drosophila*. Nature 453(7196):798-802. https://doi.org/10.1038/nature07007

Danecek P, Bonfield JK, Liddle J, Marshall J, Ohan V, Pollard MO, Whitwham A, Keane T, McCarthy SA, Davies RM, Li H (2021) Twelve years of SAMtools and BCFtools. Gigascience 10(2):giab008. https://doi.org/10.1093/gigascience/giab008

Ge, SX, Jung, D, Yao, R (2020). ShinyGO: a graphical gene-set enrichment tool for animals and plants. *Bioinformatics* 36, 2628–2629. https://doi.org/10.1093/bioinformatics/btz931

Handler D, Olivieri D, Novatchkova M, Gruber FS, Meixner K, Mechtler K, Stark A, Sachidanandam R, Brennecke J (2011) A systematic analysis of *Drosophila* TUDOR domain-containing proteins identifies Vreteno and the Tdrd12 family as essential primary piRNA pathway factors. EMBO J*.* 30(19):3977-93. https://doi.org/10.1038/emboj.2011.308

Iwasaki YW, Sriswasdi S, Kinugasa Y, Adachi J, Horikoshi Y, Shibuya A, Iwasaki W, Tashiro S, Tomonaga T, Siomi H (2021) Piwi-piRNA complexes induce stepwise changes in nuclear architecture at target loci. EMBO J 40(18):e108345. https://doi.org/10.15252/embj.2021108345

Langmead B, Salzberg SL (2012) Fast grapped-read alignment with Bowtie 2. Nat Methods 9(4):357-9. https://doi.org/10.1038/nmeth.1923

Leach TJ, Mazzeo M, Chotkowski HL, Madigan JP, Wotring MG, Glaser RL (2000) Histone H2A.Z is widely but nonrandomly distributed in chromosomes of *Drosophila melanogaster*. J Biol Chem 275(30):23267-72. https://doi.org/10.1074/jbc.M910206199

Lu W-J, Chapo J, Roig I, Abrams JM (2010) Meiotic recombination provokes functional activation of the p53 regulatory network. Science 328(5983):1278-81. https://doi.org/10.1126/science.1185640

Lucy, LB (1974) An iterative technique for the rectification of observed distributions. Astronomical Journal 79:745. doi: 10.1086/111605

Quinlan AR (2014) BEDTools: The Swiss-army tool for genome feature analysis. Curr Protoc Bioinformatics 47:11.12.1-34. https://doi.org/10.1002/0471250953.bi1112s47

Ramírez F, Ryan DP, Grüning B, Bhardwaj V, Kilpert F, Richter AS, Heyne S, Dündar F, Manke T (2016) deepTools2: a next generation web server for deep-sequencing data analysis. Nucleic Acids Res 44(W1):W160-5. https://doi.org/10.1093/nar/gkw257FormularbeginnFormularende

Richardson, WH (1972) Bayesian-based iterative method of image restoration. J. Opt Soc Am 62, 55-92. https://doi.org/10.1364/JOSA.62.000055

Robinson JT, Thorvaldsdóttir H, Winckler W, Guttman M, Lander ES, Getz G, Mesirov JP (2011) Integrative genomics viewer. Nat Biotechnol 29(1):24-6. https://doi.org/10.1038/nbt.1754

Rørth P (1998) Gal4 in the *Drosophila* female germline. Mech Dev 78(1-2):113-8. https://doi.org/10.1016/s0925-4773(98)00157-9

Shpiz S, Kwon D, Uneva A, Kim M, Klenov M, Rozovsky Y, Georgiev P, Savitsky M, Kalmykova A (2007) Characterization of *Drosophila* telomeric retroelement TAHRE: transcription, transpositions, and RNAi-based regulation of expression. Mol Biol Evol 24(11):2535-45. https://doi.org/10.1093/molbev/msm205

Shpiz S, Olovnikov I, Sergeeva A, Lavrov S, Abramov Y, Savitsky M, Kalmykova A (2011) Mechanism of the piRNA-mediated silencing of *Drosophila* telomeric retrotransposons. Nucleic Acids Res 39(20):8703-11. https://doi.org/10.1093/nar/gkr552

Stocker H, Radimerski T, Schindelholz B, Wittwer F, Belawat P, Daram P, Breuer S, Thomas G, Hafen E (2003) Rheb is an essential regulator of S6K in controlling cell growth in *Drosophila*. Nat Cell Biol 5(6):559-54. https://doi.org/10.1038/ncb995

Teo RYW, Anand A, Sridhar V, Okamura K, Kai T (2018) Heterochromatin protein 1a functions for piRNA biogenesis predominantly from pericentric and telomeric regions in *Drosophila*. Nat Commun 91(1):1735. https://doi.org/10.1038/s41467-018-03908-3

Tyanova S, Temu T, Sinitcyn P, Carlson A, Hein MY, Geiger T, Mann M, Cox J (2016) The Perseus computational platform for comprehensive analysis of (prote)omics data. Nat Methods 13(9):731-40. https://doi.org/10.1038/nmeth.3901

Vojtek AB, Hollenberg SM, Cooper JA (1993) Mammalian Ras interacts directly with the serine/threonine kinase raf. Cell 74(1):205-14. https://doi.org/10.1016/0092-8674(93)90307-c

Wickham H (2016) ggplot2, Use R! Springer International Publishing, Cham. https://doi.org/10.1007/978-3-319-24277-4.

Wickham H, Averick M, Bryan J, Chang W, McGowan LD, François R, Grolemund G, Hayes A, Henry L, Hester J, Kuhn M, Pedersen TL, Miller E, Bache SM, Müller K, Ooms J, Robinson D, Seidel DP, Spinu V, Takahashi K, Vaughan D, Wilke C, Woo K, Yutani H (2019) “Welcome to the tidyverse.” Journal of Open Source Software, 4(43), 1686. https://doi.org/10.21105/joss.01686

Other references can be found in the Reference list of the main text under the number given in parentheses.
