# Supplementary material for: putzig contributes to Piwi-mediated repression of transposon activity in the female germline of Drosophila in a two-tiered fashion to safeguard genome integrity
Source: Cell Mol Life Sci. 2026 Nov 4;83(1):410. doi: 10.1007/s00018-026-06465-x (PMC13740440; doi:10.1007/s00018-026-06465-x)
Supplement: Supplementary file 2 [file 18_2026_6465_MOESM2_ESM.docx]

**Supplemental Figures S1-S8**

**Figure S1**: **Knockdown efficiency of *pzg* in the germline**

(**A,B**) Downregulation of *white* control (UAS-shRNA-*w)* (A) and *pzg* (UAS-shRNA-*pzg*) (B) in germ cells using *mat⍺-*Gal4. Pzg protein (green) is detected in germ line cells, notably nurse cells (arrows point to examples), as well as somatic follicle cells in the control (A). In contrast, nurse cells are devoid of Pzg signal in the knock down situation cells (arrows point to examples) (B). Scale bars 20 µm. (**C,D**) Induction of control UAS-shRNA-*w* (C) and UAS-shRNA-*pzg* knockdown (D) using *nos*-Gal4. Pzg is strongly expressed in GSC nuclei in the control (C, arrows), but absent from GSCs in the *pzg* knock down (D, arrows). ⍺-Pzg, green; ⍺-Vasa, magenta as germline marker. Scale bars 10 µm. (**E**) Specific knockdown of *pzg* in germ cells using *nos*-Gal4 significantly reduces *pzg* transcripts in whole ovaries to a level of about 70%, whereas *Dref* transcripts remain unchanged. Note that *pzg* is strongly expressed in the somatic cells of the ovary, which are not affected by the knockdown. Four biological and two technical replicates were performed each. Amplification efficiencies of the four reference genes *Cpsf, cyp33, SdhA* and *Tbp* were taken into account for determining relative quantities by REST. Median corresponds to the expression ratio; mini-max depicts 95% confidence. Exact p values as determined with PFRR from REST are given above the bars.

**Figure S2**: **Inhibition of reverse transcriptase ameliorates the *pzg* mutant phenotype**

Feeding of Lamivudine (3TC), an inhibitor of reverse transcriptase, during larval development improved the atrophied phenotype of *nos*-Gal4::UAS-shRNA-*pzg* ovaries. (**A**) DAPI-stained examples for atrophied germaria (left), development of further stages up to stage 9 (about 40% of the females; middle), and germaria with occasional fully developed eggs (about 1%; right). Scale bars, 200 µm. (**B**) Statistics of 3TC feeding assay with respective controls. Number of analyzed ovaries is given above each bar; the respective genotype is indicated below. Categories are: **1 (black)**: atrophied ovaries, i.e. only structures resembling germaria; **2 (dark purple)**: rudimentary ovaries with some follicles developed up to stage 9; **3 (light purple)**: development up to stage 13 in some ovarioles; **4 (light green)**: complete egg development can be observed; **5 (dark green)**: like a wild type ovariole.

**Figure S3**: **Transcription of dual-strand piRNA clusters**

Strand-specific qRT-PCR for RNA from dual-strand clusters *38C1/C2*, *42AB* and *80F* in ovaries from freshly hatched *pzg* knock out flies (*nos*-Gal4::UAS-shRNA-*pzg)* compared to control (*nos*-Gal4::UAS-shRNA-*w*). The yellow bars show expression ratios using maternally expressed Gal4-VP16 as reference (from *nos*-Gal4-VP16). Results match the results taken from Figure 5A shown in green, where mRNA levels of the four ubiquitously expressed genes *Cpsf*, *cyp33*, *SdhA* and *Tbp* were taken as references. Relative quantities were determined by REST. Median corresponds to the expression ratio; mini-max depicts 95% confidence. All expression ratios are significant at the level of p<0.05 using PFRR from REST; exact p values are given above the bars.

**Figure S4**: **Control co-IPs and inverse co-IPs**

(**A-F**) Western Blot analysis of co-immunoprecipitations using protein lysates from ovarian extracts: proteins were captured using nano-trap, i.e. GFP-Trap® (A, D) or V5-Trap® (B), or guinea pig anti-Pzg antibody (C,E,F) as indicated. Pzg was detected with antibodies derived from rat, and the specific antibodies as shown. 10% of the Input used for the IP-traps were loaded for comparison; control describes the IP with agarose beads only.

(**A**) Unrelated control: GFP trapped from whole ovaries of flies expressing *nos*-Gal4::UASp-*GFPtubalpha*, detected with anti-GFP and anti-Pzg**. (B)** V5-trapped Pzg precipitates from ovaries harbouring additionally an EGFP-tagged Rhino transgene, detected with anti-V5 and anti-GFP. **(C)** Pzg-precipitates from ovarian extracts of Moon-GFP tagged animals, detected with anti-Pzg and anti-GFP. **(D)** GFP-trap precipitates from EGFP-Piwi tagged animals, detected with anti-Pzg and anti-GFP. **(E)** Pzg-precipitates from ovaries of Nxf2-Flag tagged animals, detected with anti-Pzg and anti-Flag. **(F)** Pzg-precipitates from ovaries detected with anti-Pzg and anti-HP1a.

**Figure S5**: **X-ChIP control data and genome wide signal correlation of Pzg with RDC members**

(**A,B**) X-ChIPs on piRNA clusters 42AB and 38C1/2 as indicated. Data from three independent experiments were averaged and normalized. Shown are ChIP-seq signals, depicted as coverage per million reads, of the Pzg IP (black), the Pzg input (grey), and the Pzg enrichment (orange) as calculated from Pzg IP versus input. Chromosomal positions are indicated below. **(C)** Control region outside piRNA clusters on chr2R with little binding of Pzg (red framed). **(D)** Genome wide ChIP-seq signal correlation of Pzg with RDC members. Shown are heatmaps of the Spearman correlation between ChIP-seq signals for Pzg, Rhi, Del and Cuff across the genome. The colour scale ranges from –1.00 (strong negative correlation, purple) to 1.00 (strong positive correlation, yellow). Data are given within the figure.

**Figure S6**: **Nuclear Piwi accumulation in GSCs depends on Pzg**

Germaria derived from 0-3 days old females, stained for Piwi (green), Pzg (blue) and Vasa (red) using the respective antibodies. (A) In the control (*nos*-Gal4::UAS-shRNA-*w*) Piwi can be detected in Vasa positive GSC nuclei (arrows). (B) Piwi nuclear accumulation is strongly reduced in *pzg*-depleted GSC nuclei (*nos*-Gal4::UAS-shRNA-*pzg*) (arrows). Scale bars, 10 µm.

**Figure S7** **Pzg’s association with factors of the piRNA processing machinery**

(**A-D**) Western Blot analysis of co-immunoprecipitations using tagged protein, GFP-Armi (A), GFP-Gasz (B), GFP-Zuc (C), or Flag-Papi (D) from ovarian extracts. Pzg was detected with specific antibodies. 10% of the Input used for the IP-traps were loaded for comparison; control describes the Trap with agarose beads only. (**E**) Yeast two-hybrid interaction assays revealed no direct protein-protein interactions between Pzg and Armi, Gasz, Zuc or Papi.

**Figure S8** **Uncropped blots of co-immunoprecipitations**

***Uncropped blots from Fig. 6A*:** Blots were sliced before antibody treatment in Rhi-, Moon-, and Trf2-trap experiments, indicated by a schematic scissor in Fig. 6A.

***Uncropped blots from Fig. 8C and F:*** Blot was sliced before antibody treatment in Piwi, indicated by a schematic scissor in Fig. 8C.

***Uncropped blots from Fig. 9A and B:***	Blot was sliced before antibody treatment in Vasa, indicated by a schematic scissor in Fig. 9B.

***Uncropped blots from Fig. 10A-D****:* Blots were sliced before antibody treatment in Mael-, Pzg-, and HP1a-trap- experiments, indicated by a schematic scissor in Fig. 10A, C, D.

***Uncropped blots from Fig. S4***: Blots were sliced before antibody treatment, indicated by a schematic scissor in Fig. S4A, B, C, F.

***Uncropped blots from Fig. S7***: Blots were sliced before antibody treatment in Gasz-trap and Zuc-trap experiments, indicated by a schematic scissor in Fig. S7B and C.
